# Commentary: Oxytocin-Gaze Positive Loop and the Coevolution of Human-Dog Bonds

**DOI:** 10.3389/fpsyg.2015.01845

**Published:** 2015-11-30

**Authors:** Sylvain Fiset, Vickie Plourde

**Affiliations:** ^1^Secteur des Sciences Humaines, Université de MonctonEdmundston, NB, Canada; ^2^École de Psychologie, Université LavalQuebec City, QC, Canada

**Keywords:** dogs, wolves, domestication, oxytocin, gaze

In recent years, it has been brought forth that dog-human relationships share several characteristics with the maternal bond that unites a child with their principal caregiver (Serpell, 2003) and that mutual gazing—sustained eye contact between two individuals (Rogers, 2013)—could be one particular way in which we express our affection or love with our dogs (Nagasawa et al., 2009). Although affection has already been the subject of many animal studies (e.g., see Harry Harlow's lifelong work with rhesus macaques by Blum, 2002), the possibility that members of two unrelated species could communicate their affection simply by looking at each other is an emerging and fascinating topic for comparative researchers (MacLean and Hare, 2015). Recently, Nagasawa et al. (2015) supported this hypothesis by revealing that oxytocin, a neuropeptide that regulates many forms of social behavior, including bonding and affiliation (Churchland and Winkielman, 2012), is largely involved in the physiological mechanisms underlying mutual gazing between dogs and humans.

In an elegant first experiment in which domestic dogs and tamed wolves were reunited for 30 min with their owners, Nagasawa et al. (2015) found that gazing behaviors from dogs, but not from wolves, increased oxytocin concentrations in owners, which in turn increased oxytocin concentrations in dogs. In addition, the increase in oxytocin in dogs and their owners correlated with the duration of dog-to-owner mutual gazing: Dogs and owners who exchanged long gazes had an increase in oxytocin, whereas oxytocin concentrations remained stable in dogs and owners that shared shorter gazes. By examining the age of acquisition of the dogs by the owners and other variables, such as the degree of socialization, the authors discarded the possible impact of an early-life experience with humans to explain these differences between dogs and wolves. Based on the results of this first experiment, Nagasawa et al. (2015) concluded that oxytocin has facilitated and modulated human-dog bonding through gazing behavior and that this particular oxytocin/mutual-gaze feedback loop possibly evolved during dog domestication. Although we encourage and applaud the efforts deployed by Nagasawa et al. (2015) to pinpoint the origins of mutual gazing between dogs and humans, we want to address a few questions regarding this particular experiment and the conclusions drawn by the authors.

Firstly, bonding in humans undeniably has roots in the relationship between a child and their primary caregiver, which is usually their mother (Feldman, 2012). In Nagasawa et al. (2015)'s study, however, the dogs' principal figure of attachment were their owners. Although dog-owner attachment behaviors are analog to child-mother's ones (Topál et al., 1998), before concluding that the oxytocin/mutual-gaze feedback loop coevolved during dog domestication, one must first examine if dog puppies and their own mothers (especially pairs of dogs that remain together for months after birth) present the same oxytocin concentrations as dogs and their owners when reunited. Indeed, even if dogs do not usually exchange mutual gazes, they can express their affection to each other through different behaviors, such as tail wagging or licking (Coren, 2000). Thus, when reunited, dogs and their mothers could present an increase of oxytocin as a function of these behaviors. If positive, this would suggest that the oxytocin feedback loop is generalized to various behaviors and to any dog's primary caregiver, whether they are human or not, casting doubt on the coevolution hypothesis.

Secondly, given that all dogs tested by Nagasawa et al. (2015) were adults, it is possible that the oxytocin/mutual-gaze feedback loop may be derived from experiences in ontogeny rather than domestication. One particular avenue to explore is the possibility that, during ontogeny, some dogs (and their owners) learn to use mutual gazing, which could be reinforced by the release of oxytocin. We therefore suggest that a similar transversal and/or longitudinal study, using a procedure similar to the one developed by Nagasawa et al. (2015), be conducted with dog puppies.

Thirdly, why did the two sub-groups of dogs (short and long gaze) respond differently to the reunion? In many species, including dogs (Romero et al., 2014), oxytocin concentrations are highly variable between members of the same species. Notwithstanding these individual differences, if the oxytocin/mutual-gaze feedback loop suggested by Nagasawa et al. (2015) coevolved during dog domestication, the same trend—an increase in oxytocin concentrations and of gaze duration before and after reunion—should be observed in all dogs, not just in long gaze dogs. Unfortunately, Nagasawa et al. (2015) did not provide a satisfying explanation for this intra-species difference. Moreover, they neglected to examine the potential effects of many important variables (e.g., age of acquisition of the dog, degree of socialization, quality of the dog-owner relationship) that could explain the differences between the short and the long gaze sub-groups.

Finally, although the authors declared that “Participants were aware of the procedure of the experiment but blind to its purpose” (p. 1, Supplementary materials), we were astonished to learn that at least one member of the research group participated in the study with his own two dogs (see Grimm, 2015). Although researchers are usually not banned from participating in their own study, it is well-known that a Rosenthal's effect can occur if participants are knowledgable about the research hypotheses, making them more inclined to behave in line with the hypotheses (Rosenthal, 1966). Thus, how many members of the research group participated in the study with their dogs? And if many did, could it be possible that their knowledge of the hypotheses increased the duration of mutual gazing, triggering the release of higher concentrations of oxytocin?

Despite the comments above, we agree that oxytocin, especially when nasally administered to dogs (see Experiment 2 in Romero et al., 2014; Nagasawa et al., 2015), appears to modulate and facilitate mutual gazing between dogs and humans. However, we strongly believe that Nagasawa et al. (2015)'s conclusion about the possible coevolution of the oxytocin/mutual-gaze feedback loop during dog domestication is premature and that further work is necessary. Until further support, we suggest that any reference to this possible coevolutionary process be presented and discussed with great care.

## Author contributions

SF drafted the article based on written comments made by SF and VP about the research report published by Nagasawa et al. (2015) in Science. Both authors contributed equally to the commentary. SF and VP reviewed numerous drafts of the article and agreed to the final version of the manuscript.

### Conflict of interest statement

The authors declare that the research was conducted in the absence of any commercial or financial relationships that could be construed as a potential conflict of interest.
